# The effect of NF-κB antisense oligonucleotide on transdifferentiation of fibroblast in lung tissue of mice injured by bleomycin

**DOI:** 10.1007/s11033-014-3273-8

**Published:** 2014-03-02

**Authors:** Yan Zhou, Xiaoye Zhang, Mingqi Tan, Rui Zheng, Li Zhao

**Affiliations:** 1 Department of Respiratory Medicine, Shengjing Hospital of China Medical University, Shenyang, China; 2 The 4th Department of Oncology, Huaxiang Branch, Shengjing Hospital of China Medical University, No. 39 Huaxiang Road, Tiexi District, Shenyan, 110004 Liaoning Province China

**Keywords:** Fibroblast, Myofibroblast, NF-κB, IkB-α, α-SMA, NF-κB antisense oligonucleotide

## Abstract

To investigate the influence of NF-κB antisense oligonucleotide on transdifferentiation of fibroblast in the pathological process of bleomycin-induced pulmonary fibrosis in mice. 6 h before molding of C57BL/6 model of pulmonary fibrosis in mice, NF-κB antisense oligonucleotide was injected from caudal vein. Then the lung tissue was collected for primary culture as well as model group and control group. Cultured cells were used for immunocytochemical staining of p65, IκB-α and α-SMA proteins as well as in situ hybridization staining of p65 and IκB-α. Then image analysis was carried out. The expressions of all the indicators were expressed as mean optical density. Compared with the control group, the expressions of p65 protein, IκB-α protein and α-SMA protein of model group were increased, as well as the expressions of p65 mRNA and IκB-α mRNA (*P* < 0.05). Compared with model group, the expressions of all indicators of intervention group were decreased (*P* < 0.05). P65 protein and p65 mRNA were positively correlated with the expression of α-SMA protein respectively. p65 protein and p65 mRNA were positively correlated with the expressions of IκB-α protein and IκB-α mRNA respectively. NF-κB antisense oligonucleotide can inhibit the transdifferentiation of fibroblast towards myofibroblast in the pathological process of bleomycin-induced pulmonary fibrosis in mice.

## Introduction

Idiopathic pulmonary fibrosis (IPF) is a disease whose pathogenesis is not clear yet. The fibroblast (Fb) attracts much attention because of its participation in the generation of extracellular matrix. The myofibroblast (MF) is a special kind of Fb. Compared with normal Fbs, the ability of MF to secrete extracellular matrix is obviously enhanced [1]. The α-smooth muscle actin (α-SMA) is considered to be the most important specific marker [2]. Currently, researches showed that, in IPE pathological process, Fbs can transform into MFs under the effects of multiple cytokines. However, the regulatory mechanism of this phenotypic transformation still remains to be further studied. Studies have shown that TGF-β1 can regulate the proliferation of smooth muscle cell and induce FB to transform into MF. Other cytokines, such as connective tissue growth factor (CTGF), interleukin-4 (IL-4), interleukin-13 (IL-13) and endothelin etc., can cooperate with TGF-β to promote MF generation or proliferation, which, therefore, leads to pulmonary fibrosis [3]. NF-κB regulates gene transcription of a variety of inflammatory factors and plays a central role in the regulation of inflammatory response. P65 is an important functional subunit of NF-κB. NF-κB exists in the cytoplasm in the non-activated state, combined with an arrestin IκB. When the cells are stimulated, IκB is rapidly degraded by protein kinase. Therefore, NF-κB is separated and activated. Activated NF-κB enters the nucleus and binds to the promoter region of target gene, which activates the target gene and its transcription.

Our previous studies have shown that the expression of p65 in macrophages significantly increases in bleomycin-induced mouse model of IPF. P65 antisense oligonucleotide not only significantly alleviates the mortality and weight loss of the test mice, but also obviously inhibits the increase of TNF-α in BALF and serum. In addition, it inhibits the formation of pulmonary fibrosis and the generation of hydroxyproline [4].

In order to investigate the effect of NF-κB/IκB on phenotypic transdifferentiation of Fb towards MF and the inbibitional effect of p65 antisense oligonucleotide on NF-κB in that transdifferentiation in pathological process of IPF, this experiment was performed. P65 antisense oligonucleotide was used for the intervention in pathological processes of bleomycin-induced pulmonary fibrosis in mice. The expressions of p65, IκB-α and α-SMA as well as their correlations in lung tissue cultured cells were studied. It was found in this research that p65 antisense oligonucleotide could be swallowed by MFs without any specific vectors (viruses, liposomes and etc.) or gene transfection and it prevented the fibrosis process and thus improved the survival rate of bleomycin-induced fibrosis in mice. It suggests that NF-κB antisense oligonucleotide may be a potential treatment of pulmonary fibrosis.

## Materials and methods

### Materials

#### Laboratory animals

C57BL/6 female mice were purchased from Beijing Vital River Laboratory Animal Technology Company, weighed from 18 to 20 g, in SPF grad.

#### Reagents

Bleomycin A2: Nippon Kayaku Co., Ltd., batch number: Y60520.

Primary antibodies were mouse anti-human p65, IκB-α and α-SMA monoclonal IgG antibodies: bought from Santa Cruz Biotechnology, Inc. The secondary antibody was biotin-labeled goat anti-mouse IgG. Immunohistochemistry kit was purchased from Beijing Zhongshan Golden Bridge Biotechnology Co., Ltd. P65 and IκB-α in situ hybridization kits: purchased from Wuhan Boster Biological Technology Co., Ltd.

## Methods

### Grouping and processing of laboratory animals

The mice were randomly divided into three groups. There were six mice in each group. Model group: bleomycin A2 (5 mg/kg, dissolved in 20 μl sterile normal saline) was instilled in trachea; control group: 20 μl of normal saline was installed in trachea. Intervention group: NF-κB antisense oligonucleotide was injected from caudal vein 6 h before modeling. Mice in model group and control group were injected with 200 μl of normal saline from caudal vein.

### Primary culture and passage of lung tissue

Three mice in each group were selected randomly on the first day after intratracheal injection as well as the third day. The mice were executed by cervical vertebra luxation. Lung tissue was collected under sterile condition and cut into approximately 2 × 2 mm tissue blocks. They were washed with PBS for three times and then spread on the bottom of culture flask, followed by primary culture at 37 °C in 5 % CO_2_. The culture medium was RPMI-1640 culture medium containing 20 % fetal bovine serum, 100 u/ml of penicillin and 100 u/ml of streptomycin. The medium was changed every 2–3 days. Cell monolayer could form around tissue blocks in 1–2 weeks. 0.25 % trypsin was used for digestion and then the cells were filled in two culture flasks for passage (1:2). Cells were passaged 3 times with this method. The 4th generation was transferred from culture flasks to six-well plates carpeted with coverslips. When the coverslips were covered with cells, various indicators could be detected.

### Identification of fibroblasts

The cultured monolayer cells were fixed with 4 % paraformaldehyde, followed by immunocytochemical staining of vimentin and cytokeratin.

### Observation of phagocytosis of NF-κB antisense oligonucleotide by fibroblasts

Fluorescently-labeled p65 antisense oligonucleotide (5′-GAAACAGATCGTCCATGGT-3′) was added to the cultured cells, followed by culture for 12 and 24 h. Then the condition of phagocytosis of NF-κB antisense oligonucleotide by Fbs was observed under inverted fluorescence microscope (OLYMPUS BX51, Japan).

### Immunocytochemical staining

The cultured monolayer cells were fixed with 4 % paraformaldehyde, followed by immunocytochemical staining of p65, IκB-α and α-SMA. Dilution ratios of primary antibodies were all 1:100. As a negative control, PBS was used instead of primary antibodies. Operations were performed according to immunohistochemistry kit steps. Diaminobenzidine (DAB) was used for developing.

### In situ hybridization staining of P65 and IκB-α

The fixative was 4 % paraformaldehyde/0.1 M PBS (PH7.2-7.6), including 1/1000 DEPC. As a negative control, PBS was used instead of the probe. The operations were performed according to in situ hybridization kit steps.

### Image acquisition and statistical analysis

5 images of each slide covered with cultured cells were taken under a microscope (400×) with microscopic image analysis system (MetaMorph/Evolution Mp 5.0/BX51;UIC/OLYMPUS,US/JP). The amount of p65, IκB-α and α-SMA were expressed as mean optical density (MOD). The mean was used for each slide. SPSS13.0 was used for statistical processing. All measurement data were expressed as x ± s and used for statistics. Total differences of mean between groups were compared by one-way ANOVA. Every two groups were compared by *t* test. Correlation analysis method was used for the statistics of correlation degree between variables. The inspection level α = 0.05.

## Results

### The comparison of p65 protein expressions in cultured cells from lung tissues of mice in the three groups

**Fig. 1:**
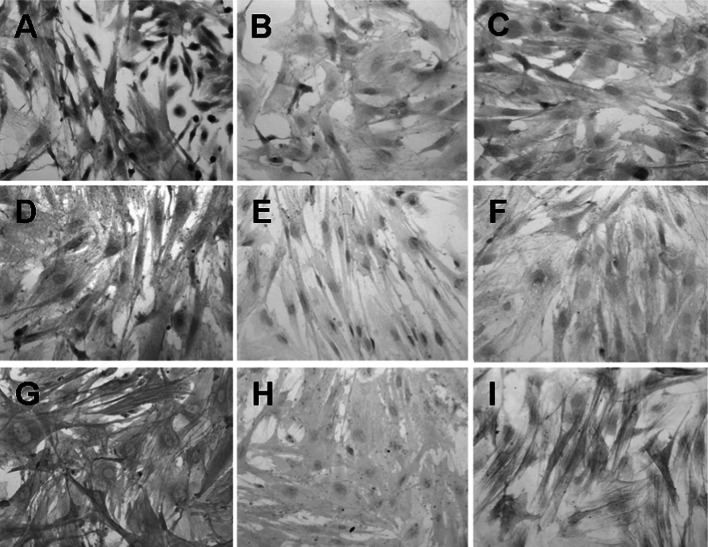
Immunocytochemical staining of p65, IκB-α, and α-SMA in model group, control group, and intervention group. **a** Immunocytochemical staining of p65 in model group. Darker staining in cytoplasm and more staining in nucleus were observed (×400). **b** Immunocytochemical staining of p65 in control group. Lighter staining in cytoplasm and rare staining in nucleus were observed (×400). **c** Immunocytochemical staining of p65 in intervention group. Compared with model group, positive staining intensity was obviously reduced; compared with control group, positive staining intensity was obviously enhanced (×400). **d** Immunocytochemical staining of IκB-α in model group. Relatively more *brownish-yellow* fine particles in cytoplasm were observed (×400). **e** Immunocytochemical staining of IκB-α in control group. A few or no *brownish-yellow* fine particle in cytoplasm was observed (×400). **f** Immunocytochemical staining of IκB-α in intervention group. Fine particles in cytoplasm were obviously less than those in model group and more than those in control group (×400). **g** Immunocytochemical staining of α-SMA in model group. Filamentous or strip darker *brownish-yellow* staining positive substance was evenly distributed in cytoplasm (×400). **h** Immunocytochemical staining of α-SMA in control group. *Pale brownish-yellow* staining in cytoplasm was observed; positive substance was obviously less than that in model group (×400). **i** Immunocytochemical staining of α-SMA in intervention group. Compared with model group, filamentous or strip *brownish-yellow* staining intensity in cytoplasm was obviously reduced; while that was obviously enhanced, compared with control group (×400). (Color figure online)

The relatively less p65 protein was expressed in cultured cells from lung tissue of mice in control group (Fig. 1b), and the MOD value was (0.0421 ± 0.0121); compared with the negative control (0.0385 ± 0.0047), the difference was not statistically significant (t = 1.734, *P* > 0.05). It shows that there is no obvious expression of NF-κB in cultured cells from lung tissue of normal mice. There was relatively more expression of p65 protein in cultured cells from lung tissue of mice in model group. The protein was distributed in the cytoplasm and (or) the nucleus and obvious positive staining was shown in the nucleus part (Fig. 1a). The MOD value was (0.1055 ± 0.0407). Compared with control group, the enhancement was significant (t = 9.449, *P* < 0.05). It indicates that NF-κB protein expression in cultured cells from lung tissue was significantly enhanced after intratracheal instillation of BLM. Compared with experimental group, p65 positive staining intensity in intervention group was obviously reduced. However, compared with control group, it was obviously enhanced (Fig. 1c). The MOD value was (0.0774 ± 0.0215). Compared with model group (t = 3.866, *P* < 0.05) and control group (t = 9.031, *P* < 0.05), the differences were significant respectively. It indicates that intravenous injection of p65 antisense oligonucleotide can obviously inhibit the increase of NF-κB expression caused by BLM, but the inhibition is not complete.

### The comparison of p65 mRNA expressions in cultured cells from lung tissues of mice in the three groups

**Fig. 2:**
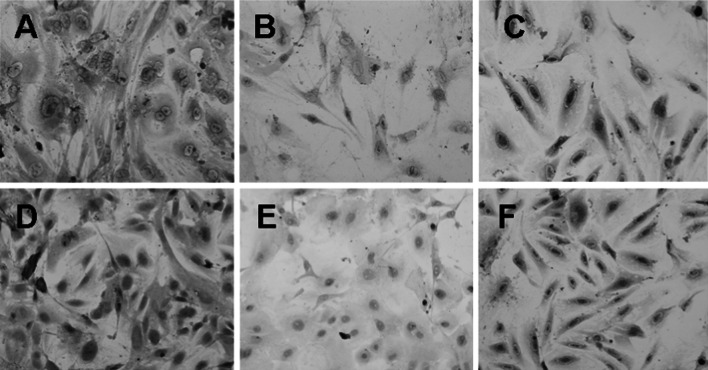
The situ hybridization staining results of p65, IκB-α expression in model group, control group and intervention group. **a** In situ hybridization staining of p65 in model group. *Darker brownish-yellow* particles in cytoplasm were observed, which showed irregular filamentous shape, massive shape or cyclic shape (×400). **b** In situ hybridization staining of p65 in control group. Part of cytoplasm was *brownish-yellow* and stained light (×400). **c** In situ hybridization staining of p65 in intervention group. Compared with model group, positive staining intensity was obviously reduced; while that was obviously enhanced, compared with control group (×400). **d** In situ hybridization staining of IκB-α in model group. There was relatively *intensive brownish-yellow* particle deposition in cytoplasm (×400). **e** In situ hybridization staining of IκB-α in control group. Compared with model group, *brownish-yellow* particles in cytoplasm were obviously reduced (×400). **f** In situ hybridization staining of IκB-α in intervention group. *Brownish-yellow* particles in cytoplasm were less than those in model group and more than those in control group (×400). (Color figure online)

The positive signal of p65 mRNA existed in the cytoplasm. Part of cytoplasm in cultured cells from lung tissue of mice in control group was pale brownish-yellow (Fig. 2b). The MOD value was (0.0613 ± 0.0135). Compared with negative control group (0.0568 ± 0.0101), the difference was not statistically significant (t = 1.674, *P* > 0.05). It indicates that there is no obvious NF-κB mRNA expression in cultured cells from lung tissue of normal mice. Compared with control group, positive signal expression of p65 mRNA in cytoplasm of cultured cells from lung tissue of mice in model group was obviously enhanced (Fig. 2a). The manifestation was claybank particles, which showed irregular filamentous shape, massive shape or cyclic shape. The MOD value was (0.1611 ± 0.0281). The difference between the two groups was statistically significant (t = 20.287, *P* < 0.05). It indicates that the expression of NF-κB mRNA in cultured cells from lung tissue was obviously enhanced after intratracheal instillation of BLM. Compared with experimental group, positive staining intensity of p65 mRNA in intervention group was obviously reduced. However, compared with control group, that was obviously enhanced (Fig. 2c). The MOD value was (0.1003 ± 0.0178). Compared with model group (t = 11.584, *P* < 0.05) and control group (t = 11.048, *P* < 0.05), the differences were statistically significant, respectively. It indicates that intravenous injection of p65 antisense oligonucleotide can obviously inhibit the increase of NF-κB mRNA expression caused by BLM, but the inhibition is not complete.

### The comparison of IκB-α protein expressions in cultured cells from lung tissues of mice in the three groups

The positive expression of IκB-α protein existed in the cytoplasm. There was a little or no brownish-yellow fine particle deposition in the cytoplasm of lung Fbs of mice in control group cultured in vitro. There was an extremely weak expression or no expression of IκB-α protein (Fig. 1e).The MOD value was (0.0342 ± 0.0034). Compared with negative control (0.0327 ± 0.0045), the difference was not statistically significant (t = 1.740, *P* > 0.05). That indicates that there is no obvious expression of IκB-α protein in cultured cells from lung tissue of normal mice. There were relatively more fine particles in the cytoplasm of lung Fbs of mice in model group (Fig. 1d). The MOD value was (0.0886 ± 0.0054). Compared with control group, the difference was statistically significant (t = 53.867, *P* < 0.05). That indicates that IκB-α protein expression in cultured cells from lung tissue was obviously enhanced after intratracheal instillation of BLM. Compared with experimental group, positive staining intensity in intervention group was obviously reduced. However, compared with control group, that was obviously enhanced (Fig. 1f). The MOD value was (0.0614 ± 0.0032). Compared with model group (t = 27.243, *P* < 0.05) and control group (t = 36.824, *P* < 0.05), the differences were statistically significant, respectively. It indicates that intravenous injection of p65 antisense oligonucleotide can obviously inhibit the increase of IκB-α protein expression caused by BLM, but the inhibition is not complete.

### The comparison of IκB-α mRNA expressions in cultured cells from lung tissues of mice in the three groups

The results from in situ hybridization detection showed that relatively intensive brownish-yellow particle deposition existed in the cytoplasm of lung Fbs of mice in model group (Fig. 2d). The MOD value was (0.1525 ± 0.0239). Obviously less brownish-yellow particles existed in the cytoplasm of lung Fbs in control group (Fig. 2e). The MOD value was (0.0658 ± 0.0146). The difference between the two groups was statistically significant (t = 19.579, *P* < 0.05). That indicates that IκB-α mRNA expression in cultured cells from lung tissue was obviously enhanced after intratracheal instillation of BLM. The difference between control group and negative control group (0.0634 ± 0.0110) was not statistically significant (t = 0.861, *P* > 0.05). That indicates that there is no obvious IκB-α mRNA expression in cultured cells from lung tissue of normal mice. Compared with experimental group, IκB-α mRNA positive staining intensity in intervention group was obviously reduced. However, compared with control group, that was obviously enhanced (Fig. 2f). The MOD value was (0.1126 ± 0.0181). Compared with model group (t = 8.047, *P* < 0.05) and control group (t = 12.715, *P* < 0.05), the differences were statistically significant, respectively. That indicates that intravenous injection of p65 antisense oligonucleotide can obviously inhibit the increase of IκB-α mRNA expression caused by BLM, but the inhibition is not complete.

### The comparison of α-SMA protein expressions in cultured cells from lung tissues of mice in the three groups

The immunocytochemical staining of α-SMA in cultured cells from lung tissue of mice in control group showed obvious positive expression. The manifestation was that filamentous or strip pale brownish-yellow α-SMA expression positive substance was evenly distributed in the cytoplasm (Fig. 1h). The MOD value was (0.0661 ± 0.0023). Compared with negative control (0.0368 ± 0.0024), the difference was statistically significant (t = 55.850, *P* < 0.05). It was observed that the number of positively stained cells in model group obviously increased and staining intensity was significantly enhanced compared with the control group (Fig. 1g). The MOD value was (0.1341 ± 0.0154). Compared with control group, the difference was statistically significant (t = 27.691, *P* < 0.05). That indicates that there is certain expression of α-SMA in lung tissue of normal mice. The expression of α-SMA in cells of lung tissue was obviously enhanced after intratracheal instillation of BLM. It was observed that positively stained cells in intervention group was stained lighter (Fig. 1i). The MOD value was (0.0985 ± 0.0195). Compared with model group, the difference was statistically significant (t = 9.075, *P* < 0.05). Compared with control group, the difference was also statistically significant (t = 10.424, *P* < 0.05). That indicates that p65 antisense oligonucleotide obviously inhibits the increase of α-SMA expression caused by BLM, but the inhibition is not complete.

### Correlations among expressions of p65 protein, IκB-α protein, α-SMA protein, p65 mRNA and IκB-α mRNA (correlation analysis of the mean of the groups)

The p65 protein was positively correlated with IκB-α protein expression (r = 0.998, *P* < 0.05); p65 protein was positively correlated with α-SMA protein expression (r = 0.971, *P* < 0.05); p65 mRNA was positively correlated with α-SMA protein expression (r = 0.955, *P* < 0.05); p65 mRNA was positively correlated with IκB-α mRNA protein expression (r = 0.989, *P* < 0.05); the correlation between IκB-α protein and α-SMA protein was not statistically significant (r = 0.826, *P* > 0.05).

### The observation of phagocytosis of NF-κB antisense oligonucleotide by lung Fbs

**Fig. 3:**
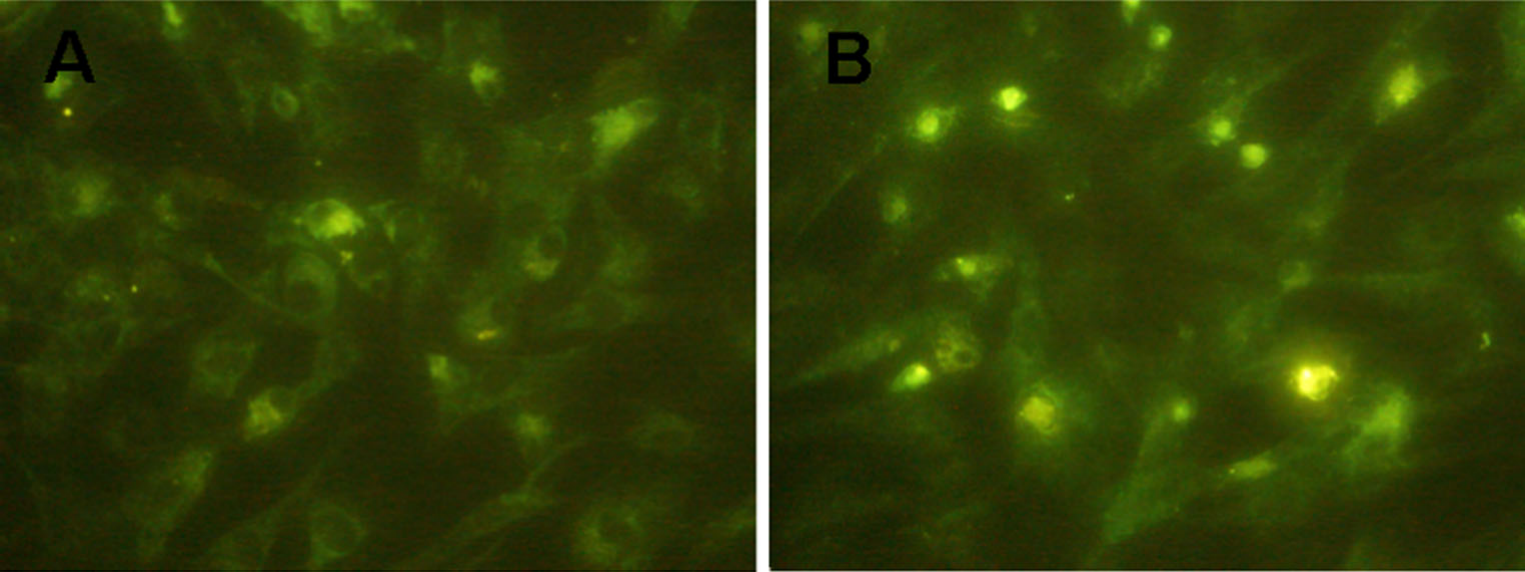
The observation of phagocytosis of p65 antisense oligonucleotide by fibroblasts. **a** Fluorescently-labeled p65 antisense oligonucleotide was added to cultured cells, followed by culture for 12 h. Obvious fluorescent developing in fibroblasts was observed under fluorescence microscope, mainly in the cytoplasm. **b** After the culture for 24 h, fluorescent developing was also observed in nuclei of part of the fibroblasts

The fluorescently-labeled p65 antisense oligonucleotide was added to cultured cells, followed by culture for 12 h. Obvious fluorescent developing was observed in Fbs under fluorescence microscope, mainly in the cytoplasm (Fig. 3a). After the culture for 24 h, fluorescent developing was also observed in the nuclei of part of Fbs (Fig. 3b).

## Discussion

Pulmonary fibrosis (PF) is a disease whose pathogenesis is not clear. It lacks an effective treatment. In recent years, studies have shown that pulmonary fibrosis had a process of the transformation of pulmonary interstitial Fbs into MFs accompanied by a large quantity of extracellular matrix deposition. The appearance and persistence of MFs played a key role in the formation of PF [5]. At present, the regulatory mechanism of the transformation of MFs has not been clear yet.

In order to investigate the effect of NF-κB on the phenotypic transformation of lung Fbs in the pathological process of bleomycin-induced pulmonary fibrosis and research the preventive effect of NF-κB antisense oligonucleotide on bleomycin-induced pulmonary fibrosis in mice, primary culture of lung tissues of mice in pulmonary fibrosis model group and NF-κB antisense oligonucleotide treatment group was performed. And immunocytochemical staining and in situ hybridization technology were used. In addition, the expressions of NF-κB in Fbs in various groups were compared and the relationship among NF-κB, IkB-α and α-SMA was studied. In this experiment, tissue explants adherent method was used for primary culture of lung Fbs. After 4 generations, immunocytochemical staining showed that the expression of vimentin was strongly positive and the expression of keratin was negative. Therefore, the cells were identified as Fbs. The cultured cells could guarantee the reliability and repeatability of the experiment (Tables 1, 2).

**Table 1:** Mean optical density values in the groups with immunocytochemical staining and in situ hybridization staining (mean ± SD)

Group	*n*	p65 protein	IκB-α protein	α-SMA protein	p65 mRNA	IκB-α mRNA
Model group	40	0.1055 ± 0.0407	0.0886 ± 0.0054	0.1341 ± 0.0154	0.1611 ± 0.0281	0.1525 ± 0.0239
Intervention group	40	0.0774 ± 0.0215	0.0614 ± 0.0032	0.0985 ± 0.0195	0.1003 ± 0.0178	0.1126 ± 0.0181
Control group	40	0.0421 ± 0.0121	0.0342 ± 0.0034	0.0661 ± 0.0023	0.0613 ± 0.0135	0.0658 ± 0.0146
Negative control group	40	0.0385 ± 0.0047	0.0327 ± 0.0045	0.0368 ± 0.0024	0.0568 ± 0.0101	0.0634 ± 0.0110
F		70.289	1565.895	447.445	268.981	234.144
*P*		<0.05	<0.05	<0.05	<0.05	<0.05

**Table 2:** The correlation coefficients among the mean expressions

Protein	IκB-α protei	α-SMA protein	IκB-α mRNA
p65 protein	0.998^#^	0.971^#^	ND
IκB-α protein	ND	0.826	ND
p65 mRNA	ND	0.955^#^	0.989^#^

^#^ represents *P* < 0.05

NF-κB is an important transcription factor and P65 is the main functional subunit of NF-κB [6]. Non-activated NF-κB exists in the cytoplasm. When activated, NF-κB enters the nucleus and binds to the promoter region of the target gene, which activates the target gene and begins its transcription.

Lung Fbs can synthesize and secrete a variety of cytokines. Besides, they can synthesize collagen and matrix proteins. By the secretion of those cytokines, Fbs form an inflammatory reaction self-excited positive feedback, which aggravates and extends the disease duration of fibrosis [7]. After the transformation of Fbs into MFs, the synthesis and secretion abilities are obviously enhanced. Meanwhile, the MF has contractile characteristic, which may change the mechanical characteristics of fibrotic lung tissue and reduce the compliance of lung. That characteristic is related to the increase of α-SMA expression [8, 9]. That whether NF-κB affects the transformation of Fbs into MFs in the process of BLM-induced lung injury has not been reported yet. The Fb can synthesize and secrete various cytokines, thus it is inferred that the expressions of NF-κB and α-SMA in the Fb may increase in BLM-induced lung injury. This experiment confirms that hypothesis. In this research, it was found that the expressions of NF-κB protein and NF-κB mRNA in primarily cultured lung Fbs of mouse model of pulmonary fibrosis were obviously enhanced, compared with normal mice. That suggests that NF-κB may be involved in the process of the synthesis of certain cytokines in lung Fbs. It was also found that the number of α-SMA positive cells in lung Fbs of mice with pulmonary fibrosis cultured in vitro was obviously more than that of normal mice. That indicates that the increase of MFs may be closely related to PF. Therefore, it is inferred that NF-κB expressed in interstitial cells may achieve its pathological function by regulating the transformation of fibroblasts into MFs, in the process of BLM-induced lung injury in mice and the following formation of pulmonary fibrosis.

Firstly, in this experiment, it was verified that the expression of α-SMA in pulmonary interstitial cells was obviously increased in the process of BLM-induced lung injury, which was similar to other research conclusions [8, 10]. Though, it has not been reported so far that NF-κB directly played a role in the regulation of α-SMA synthesis, there might be a close relationship between them. TNF-α, TGF-β, IL- 1β and PDGF etc. can activate lung Fbs and have chemotactic effect on lung Fbs. In addition, they can promote the mitosis and stimulate the collagen synthesis of lung Fbs [11, 12]. When activated, NF-κB is involved in transcriptional regulation of gene expressions of a variety of cytokines and inflammatory mediators [13] and the stimulation of lung Fb hyperplasia [14]. It was also found in the experiment that p65 protein (*r* = *0.971, P* < *0.05*), the subunit of NF-κB, and p65 mRNA (*r* = *0.955, P* < *0.05*) were both positively correlated with the expression of α-SMA protein in lung Fbs in the process of bleomycin-induced acute lung injury. According to that, it is inferred that NF-κB may promote the transformation of Fbs into MFs by some way to promote pulmonary fibrosis in the pathological process of pulmonary fibrosis.

Possible mechanism of NF-κB’s promotion of the transformation of lung Fbs into MFs: After the action of internal and external stimulation on cells, NF-κB is activated and then enters the nucleus to regulate the transcription of responsive genes of NF-κB [15]. Many genes encoding inflammatory molecules and proliferative factors are the responsive genes of NF-κB. TGF-β1 is the most important growth factor which induces MF phenotype [16, 17]. Researches at home and abroad confirm that exogenous TGF-β1 can induce the differentiation of human embryonic lung cells into the MF phenotype, and time and dose–effect relationship exists [18]. By in vitro experiment, Vancheri et al. [19] also confirm that TGF-β1, IL-4, IL-13 and bradykinin etc. can induce the differentiation of lung Fbs into MFs. Some growth factors (such as PDGF,IGF-1 and FGF) can lead to proliferative phenotype of lung Fbs and fibrosing phenotype eventually, which results in the differentiation of lung Fbs into MFs. NF-κB promotes the transformation of lung fibroblasts into MFs by regulating the transcription of various chemokines, adhesion molecules and growth factors. Myofibroblasts can produce extracellular matrix and secrete cytokines, growth factors and inflammatory mediators. Therefore, MFs play an important role in the formation of pulmonary fibrosis.

In order to further investigate the effect of NF-κB on the phenotypic transformation of Fbs in the process of BLM-induced lung injury, activation of NF-κB was blocked by injection of P65 antisense oligonucleotide from caudal vein 6 h before intratracheal instillation of BLM. The results showed that P65 antisense oligonucleotide obviously inhibited the increase of P65 expression caused by BLM in pulmonary interstitial cells. In addition, α-SMA expression was also obviously inhibited. That further confirms our speculation, that is, NF-κB may regulate the transformation of Fbs into MFs through some indirect relationship and this regulation may play an important role in the pathogenesis of pulmonary fibrosis.

The activation of NF-κB is regulated by precise mechanisms. Regulation of NF-κB activation includes positive and negative feedback regulation. Positive feedback regulation occurs mainly in the extracellular domain, which is the extracellular mechanism of the amplification of inflammatory reaction [20, 21]. Negative feedback regulation can inhibit further activation of NF-κB and limit the continuous expansion of inflammatory reaction [22, 23]. IκB-α is a super inhibitor of NF-κB. Inhibiting the degradation of IκB-α or increasing its synthesis can obviously inhibit the activity of NF-κB [24]. Griesenbach et al. [25] transfect airway epithelial cells with adenovirus carrying IκB gene, and they find that directly increasing the expression of IκB in the body can inhibit the activation of NF-κB and block the synthesis of inflammatory factors regulated by NF-κB. Then the occurrence of lung inflammation is controlled and ALI is cured. That whether IκB-α played a role in negative feedback regulation of NF-κB activation has not been reported by relevant literature yet. It is inferred by us that IκB-α will increase with the activation of NF-κB and then inhibit the further activation of NF-κB. Experiment results confirm that speculation. It was found in this research that the expression of IκB-α in cultured lung Fbs of model mice was obviously more than that of normal mice. When the activation of NF-κB was blocked by P65 antisense oligonucleotide, IκB-α expression decreased. That suggests that IκB-α’s negative feedback inhibition also weakens with the weakening of NF-κB activation. Correlation analysis of the indicators was performed. And, it was found that the mean of NF-κBp65 of all groups was positively correlated with the means of IκB-α protein expression and mRNA expression of all groups in primarily cultured lung Fbs. That suggests that after activation, NF-κB can upregulate the expression of IκB-α gene. The increase of IκB-α expression can prevent the excessive increase of NF-κB to achieve negative feedback regulation. It is found in our previous studies [2] that the expression of NF-κBp65 in lung Fbs of model mice with pulmonary fibrosis cultured in vitro reaches a peak at 24 h and it gradually decreases after that. It is considered that the result is closely related to IκB-α’s negative feedback regulation.

The effects of NF-κB antisense oligonucleotide on the survival of mice with bleomycin-induced pulmonary fibrosis and phenotypic transformation of cultured Fbs were studied. In model group, intratracheal instillation of bleomycin A2 (5 mg/kg) was performed, followed by observation for 2 weeks. The mortality rate was 20 % (6/30). In intervention group, NF-κB antisense oligonucleotide was injected from caudal vein 6 h before modeling. No mice died. It was visible that antisense oligonucleotide significantly improved the mouse survival rate. The weight of mice with bleomycin-induced pulmonary fibrosis obviously increased after intervention of antisense oligonucleotide. It was observed under fluorescence microscope that there was a large quantity of fluorescently-labeled NF-κB antisense oligonucleotide in cultured lung Fbs. That certified that NF-κB antisense oligonucleotide can enter Fbs by phagocytosis without any specific vector (viruses, liposomes and etc.) or gene transfection. Through immunocytochemical staining, it was further confirmed that antisense oligonucleotide could inhibit NF-κB, activate the Fbs and inhibit the transdifferentiation of Fbs into MFs. Those results indicate that endocytosis of antisense oligonucleotide by activated Fbs can alleviate acute lung injury/fibrosis and thus improve the survival situation of mice with bleomycin-induced pulmonary fibrosis.

Currently, the main drugs for treatment of pulmonary fibrosis are corticosteroids and/or immunosuppressants. Therapeutic effects are limited and side effects are relatively serious. The advantages and disadvantages should be weighed, when the drugs are used for treatment. Various attempts are performed on animal models, hoping to find a more effective drug for treatment of pulmonary fibrosis. In our experiment, no specific vector was used. Antisense oligonucleotides entered the activated lung Fbs or were swallowed by activated lung Fbs. Those antisense oligonucleotides not only prevented the bleomycin-induced pulmonary fibrosis, but also might be effective on other kinds of pulmonary fibrosis, including IPF. Our studies show that NF-κB plays an important role in the pathological process of pulmonary fibrosis. NF-κB antisense oligonucleotide can inhibit bleomycin-induced acute lung injury/fibrosis. Therefore, it may become an effective way of gene therapy. Through animal experiments, it is primarily proved by us that NF-κB antisense oligonucleotide can inhibit the transdifferentiation of Fbs into MFs and cure pulmonary fibrosis. However, specific mechanism, signal transduction pathways, clinical safety and effectiveness need to be further confirmed. It is believed that with the research of related pathogenesis of immunology and molecular biology, gene therapy will become a safe and effective treatment for pulmonary fibrosis.
